# Contribution of Visual Information about Ball Trajectory to Baseball Hitting Accuracy

**DOI:** 10.1371/journal.pone.0148498

**Published:** 2016-02-05

**Authors:** Takatoshi Higuchi, Tomoyuki Nagami, Hiroki Nakata, Masakazu Watanabe, Tadao Isaka, Kazuyuki Kanosue

**Affiliations:** 1 Faculty of Sport Sciences, Waseda University, Tokorozawa, Japan; 2 Research Organization of Science and Engineering, Ritsumeikan University, Kusatsu, Japan; 3 Faculty of Human Life and Environment, Nara Women’s University, Nara, Japan; 4 Faculty of Sports Science, Fukuoka University, Fukuoka, Japan; 5 Faculty of Sport and Health Science, Ritsumeikan University, Kusatsu, Japan; VU University Amsterdam, NETHERLANDS

## Abstract

The contribution of visual information about a pitched ball to the accuracy of baseball-bat contact may vary depending on the part of trajectory seen. The purpose of the present study was to examine the relationship between hitting accuracy and the segment of the trajectory of the flying ball that can be seen by the batter. Ten college baseball field players participated in the study. The systematic error and standardized variability of ball-bat contact on the bat coordinate system and pitcher-to-catcher direction when hitting a ball launched from a pitching machine were measured with or without visual occlusion and analyzed using analysis of variance. The visual occlusion timing included occlusion from 150 milliseconds (ms) after the ball release (*R+150*), occlusion from 150 ms before the expected arrival of the launched ball at the home plate (*A-150*), and a condition with no occlusion (*NO*). Twelve trials in each condition were performed using two ball speeds (31.9 m·s^-1^ and 40.3 m·s^-1^). Visual occlusion did not affect the mean location of ball-bat contact in the bat’s long axis, short axis, and pitcher-to-catcher directions. Although the magnitude of standardized variability was significantly smaller in the bat’s short axis direction than in the bat’s long axis and pitcher-to-catcher directions (*p* < 0.001), additional visible time from the *R+150* condition to the *A-150* and *NO* conditions resulted in a further decrease in standardized variability only in the bat’s short axis direction (*p* < 0.05). The results suggested that there is directional specificity in the magnitude of standardized variability with different visible time. The present study also confirmed the limitation to visual information is the later part of the ball trajectory for improving hitting accuracy, which is likely due to visuo-motor delay.

## Introduction

Interceptive actions in sports, such as hitting or blocking a target, require accuracy in both decision-making and body movement. In baseball, once a decision to swing is made, the batter accelerates the barrel end of a 0.9 kg (31.75 ounces) bat to a speed of approximately 31 m·s^-1^ (70 miles per hour [MPH]) during the final 300 ms or less [1]. For professional batters, the length of time from the initiation of bat acceleration to ball-bat contact is approximately 180 ms [2]. In addition, the optimal way to hit a pitched ball is with the small “sweet spot” of a bat. The deviation of ball-bat contact from the sweet spot in bat’s long axis direction causes greater vibration in the bat, which results in a lower batted ball speed, whereas the deviation of ball-bat contact in a bat’s short axis direction increases the chance for ground out or fly out [3]. Therefore, batters need to hit a ball within an acceptable range of contact area in the bat’s long and short axis directions while a bat is moving at high speed. One reason why hitting a baseball is considered “one of the most difficult skills in sports [4]” is the difficulty in identifying the arrival time and location of a pitched ball before the batter becomes unable to change the motion of the swung bat. Therefore, batters with a good success rate (batting average ≥ 0.300) can be categorized as elite players.

The latency between visual perception and muscle contraction, termed the visuo-motor delay, is a limiting factor for making visual information available to guide the bat swing in the process of hitting a pitched baseball [5]. The visuo-motor delay varies depending on the type of task. Simple correction based on movement of the target, such as pointing at a moving target that has an unexpected acceleration [6,7] or moving the hand in the same direction as a target is moving [8] takes approximately 150 ms to 200 ms. Furthermore, some investigators report that tasks requiring large-scale corrections, such as aiming at a moving target with an unexpected change in direction and speed [9] or decelerating a bat’s motion against a non-strike ball [10] may require 200 ms to 250 ms. Visual reaction time in a hitting task during a game of cricket was approximately 200 ms for both professional and nonprofessional cricket players [11]. Since the flight time of a pitched ball from the pitcher’s mound to the home plate is approximately 400 ms to 500 ms, the visual information gained from the second half or last one-third of the pitch trajectory may not contribute to adjusting the swing motion. However, “keeping one’s eyes on the ball” is believed to be helpful for improving hitting accuracy by some coaches and players [12].

The accuracy in prediction and decision-making will be improved if the batter can see the pitched ball for a longer time, but the closer the ball travels, the less time is available for the batter to swing the bat or adjust the trajectory of the swung bat. For experienced batters, it is possible to determine a standard trajectory for a fastball based on its speed, because there is a correlation between the speed and backspin rate (which are the major determinants of the trajectory of a pitched ball [13]) of fastballs thrown by actual pitchers [14,15]. In fact, experienced batters tended to mishit by swinging under the ball more than usual when the backspin rate of a launched fastball was greater than the typical range of backspin rates for a given ball speed, which results in less drop of the ball during its flight [16] In addition, previous studies utilizing a virtual baseball hitting task found that experienced batters have superior “strike-zone sensitivity,” which is the ability to make correct decisions to swing at only strikes and not balls, and shorter reaction time for pitch recognition compared with less experienced batters [17,18]. Fadde [19] confirmed that two weeks of pitch recognition training using videos of pitches that were occluded after a certain time after ball release is helpful for improving batting average. Therefore, batters seem to have acquired the ability to accurately predict the arrival location of a pitch through years of hitting experience.

Although predicting the arrival location of a pitched baseball seems to be a crucial component of accurate hitting, the relationship between actual baseball hitting accuracy and visual information is still unclear. A study conducted by de Lucia and Cochran [20] reported that screening the first third, middle third, or last third of the ball trajectory decreases the percentage of ball-bat contacts and the timing accuracy. However, their study used only a relatively slow ball speed (26.7 m/s; 60 MPH), and a screen was placed between the batter and a tennis-ball serving machine to hide the launched ball, which may have resulted in inconsistent availability of visual information due to the batter’s head motion. One way to control visual information about the pitch trajectory is to occlude the batter’s eyesight at different times following the ball launch while the batter attempts to hit the ball. Some research does exist that examines hitting accuracy with occluded vision by establishing discrete data, though these data were collected using a different sport. Müller and Abernethy [21] reported that elite cricket batsmen were able to hit a thrown ball 60% of the time when their vision was occluded from the moment of ball release. Moreover, they found no significant difference in the percentage of ball-bat contact between the trials with visual occlusion beginning at the moment of ball bounce and trials without visual occlusion. Other research on cricket batting reported that national-level cricket batsmen were able to correctly identify which of five possible pitches was delivered 63% of the time even though their vision was occluded 80 ms after ball release [22]. Therefore, the ability to utilize the early part of the ball’s trajectory seems to play a key role in hitting accuracy by experienced batters.

In the present study, we examined the relationship between hitting accuracy and the segment of the trajectory of the flying ball that can be seen by a batter during actual batting. To identify the amount of spatial error, which has different effects along the bat’s long axis and short axis, and on the temporal error, we focused on the ball-bat contact location on the bat’s surface and the pitcher-to-catcher direction. The influence of visible time on the spatial and temporal components of hitting accuracy were evaluated using the mean location of ball-bat contact, which represents the amount of systematic error and standardized standard deviation (SD) for ball-bat contact with the SD for the arrived ball location, which represents standardized variability. Previous studies on elite batters have reported their superb ability to use the early part of the ball trajectory to successfully hit the ball. Moreover, it seems that it would be difficult to correct the trajectory of a moving bat once the bat has head reached a certain speed. Therefore, we hypothesized that a longer visible time improves the hitting accuracy except visual information about the 150-ms period before ball-bat contact, because this is approximately the time required to react to a visual stimulus [6–10], additionally, it is the length of time from the initiation of bat acceleration to ball-bat contact [2]. Since the length of the ball trajectory that can be seen and the amount of time left to complete bat swing differs by ball speed at a given visible time, two hitting tasks, one with a relatively fast ball speed and another with a relatively slow ball speed in the actual baseball game, were utilized.

## Materials and Methods

The experimental procedures, risks, and benefits were explained to all participants before recordings were taken. Then written consent was obtained from all participants. All experimental procedures received approval from the Ethics Review Committee on Human Research of Waseda University (approval number: 2010043).

### Participants

Ten collegiate baseball field players (five right handed batters and five left handed batters) participated in the experiment. The mean ± SD age, height, and body mass of the study participants were 20.2 ± 1.1 years, 1.78 ± 0.05 m, and 76.3 ± 6.6 kg, respectively. The mean length of their baseball experience was 11 ± 1.2 years (range: 9–13). Participants performed the experiment in one day and were instructed to eat and drink water normally on the day of and one day before the experiment. In addition, they were told to sleep normally the night before the experiment, and each participant was asked about their physical and mental state before the recording; all responses were good or normal.

### Apparatus

An arm-style pitching machine (NA61, Nisshin SPM Ltd., Beppu, Japan) was used for this study. Its launch point was located 17 m (55.77 feet) from the home plate and 1.6 m (5.25 feet) from the ground. The pitching machine was set to launch a ball with backspin, to replicate a fastball trajectory. To reduce the risk of injury if hit by a pitch and minimize pain to the batter’s hands due to mishits, polyurethane balls (diameter = 74 mm [2.91 inches], mass = 125 g [4.41 ounces], Mizuno Co., Osaka, Japan) that are manufactured for baseball hitting practice were utilized. Participants were given the choice of four types of wooden bats (length = 840 mm [33.01 inches], mass = 900 g [31.75 ounces]). Participants wore a visual occlusion liquid-crystal apparatus (PLATO, Translucent Technologies). The timing of vision occlusion was controlled by a software program (ToTaL control 2.0, Translucent Technologies), which initiated occlusion at the chosen time after a launched pitch caused a trigger signal to be sent from a photo-sensing diode (PLDM-10, Sankei Machinery, Tokyo, Japan) mounted on the pitching machine’s ball release point.

### Procedure

After sufficient warm-up and practice hitting, the participants completed five hits off of a batting tee using maximal effort to measure their bat swing speed. Then they completed two sets of 36 hits from the pitching machine. One of two ball speed settings was used throughout the first set, and the other speed setting was used for the second set. For the slow ball setting, the ball speed was set at 31.9 m·s^-1^ (115 km·h^-1^; 71.8 MPH). For the fast ball setting, the ball speed was set at 40.3 m·s^-1^ (145 km·h^-1^; 90.7 MPH). The order of the two speed settings was randomized for each participant and counterbalanced across all participants. The ball speeds were observed by a radar gun (The Jugs Company Japan Ltd., Higashi-Osaka, Japan) during the trials to ensure consistency. The spin axis of the launched ball was similar to the spin axis of a pitch with true backspin. The average ball spin rates from 10 trials were determined before the study began using a high-speed video camera (Frame rate = 1000 Hz, exposure time = 0.5 ms, resolution = 680 × 480 pixels; Trouble Shooter, Fastec Imaging Corporation, San Diego, USA). The average spin rates were 30.6 rotations per second for the slow ball setting and 36.1 rotations per second for the fast ball setting. Both sets consisted of 12 hits under each of three different conditions: no occlusion (*NO*), occlusion beginning 150 ms after the ball release (*R+150*), and occlusion beginning 150 ms before the expected arrival of the launched ball at the home plate (*A-150*). Participants were not aware of the type of occlusion to be performed. The expected arrival time of the ball at the home plate was determined before the study began by using the photo-sensing diode, which detects the ball release, and a sound-to-electrical transducer trigger unit (ATRG-100, Nihon Fastec Imaging Co. Ltd., Tokyo, Japan), which detects the sound of a ball hitting an object located on the home plate. Signals from the two sensors were transferred to a data acquisition system (PowerLab, AD Instruments Japan) to calculate the length of flight time. In the experimental trials, one of three occlusion conditions was randomly assigned in a counter-balanced sequence and was initiated by a signal from the photo-sensing diode that detects the ball launch.

To avoid participants being hit by a launched ball, balls were set to launch towards the strike zone above the outer half of the home plate away from the batter. The ball height was randomly altered by changing the anterior-posterior tilt angle setting of the pitching machine in every trial. Participants were not aware of this process and were instructed to place their trailing (back) foot at their preferred position and to maintain the same foot position during all trials. Participants were instructed to try to hit the ball with the part of the barrel that was marked, which was located 150 mm (5.91 inches) from the barrel end of the 840 mm bat. In the present study, this part was considered the “sweet spot” of the bat, because this impact point is located between two nodal points of vibration (170 mm and 130 mm from the barrel end) for a 840 mm wood bat [23,24].

### Data collection

Bat and ball movement was recorded using two synchronized high-speed video cameras. One camera was placed 6 m away from the home plate at a right angle to a line between the center of the pitching rubber and the center of the home plate, and the other camera was placed 6 m behind the home plate to provide a rear view of the hitting movement. The hitting motion was recorded for 150 ms before and 150 ms after a sound-to-electrical transducer trigger unit registered the ball-bat contact. Reflective tape was attached to the barrel end of the bat and 450 mm down from the barrel end of the bat to aid data analysis.

### Data analysis

**Fig 1:**
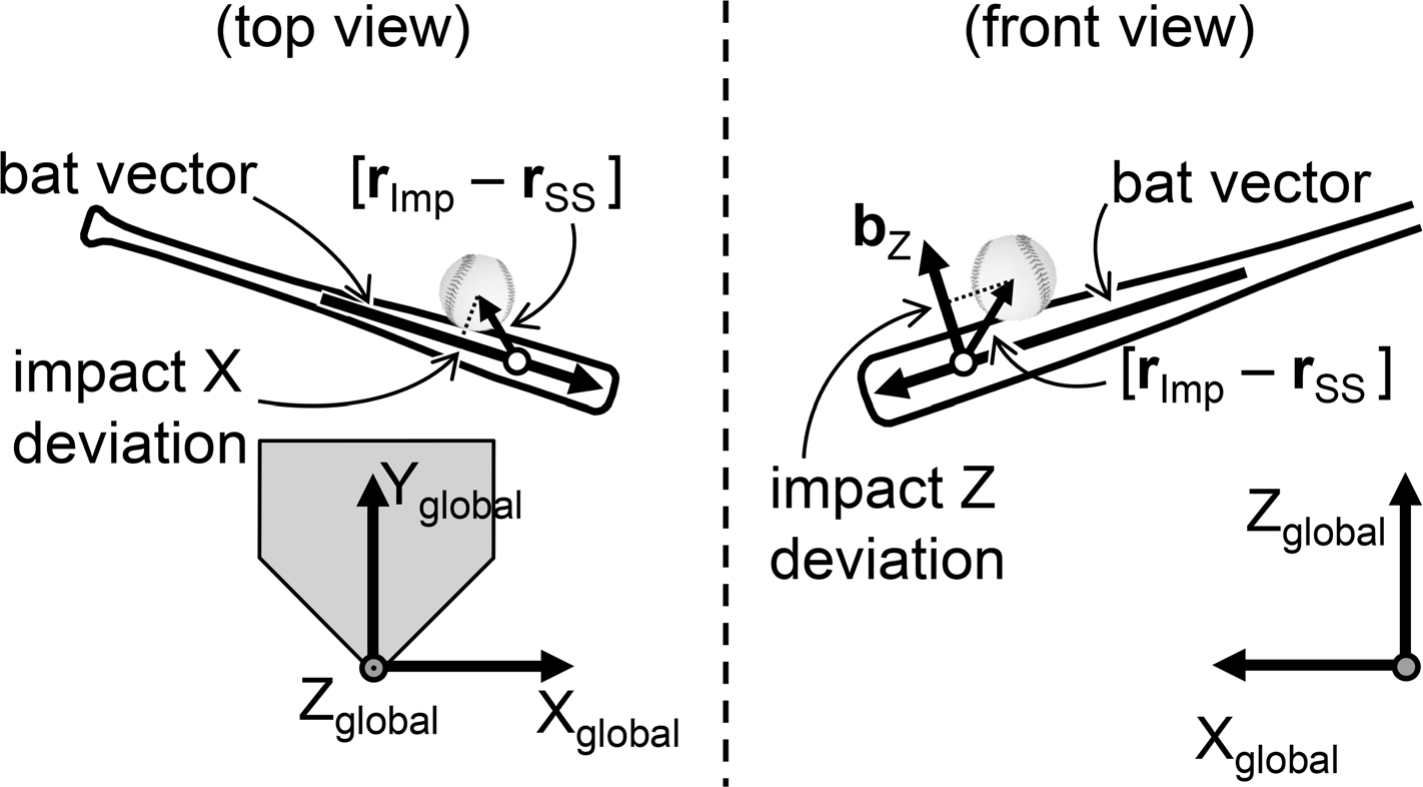
Definition of the global coordinate system and the bat coordinate system. The drawing represents the instant of ball-bat contact from the top view (left) and front view (right). Impact X deviation is the distance between the sweet spot of the bat (white circle; r_ss_) and the center of the ball (r_Imp_) in the direction of bat vector. Impact Z deviation is the distance between the sweet spot of the bat (white circle; r_ss_) and the center of the ball (r_Imp_) in the direction of b_z_.

Data involving the location of the bat top, the bat grip, and the launched ball were obtained from the images, digitized, and analyzed using the Frame Dias IV motion analysis system (DKH, Tokyo, Japan). Five frames (5 ms) immediately before the frame that captured the ball-bat contact were utilized for the analysis of bat swing speed. One frame (1 ms) before the frame that captured the ball-bat contact was utilized for the analysis of ball-bat location. Three-dimensional coordinates were obtained using the direct linear transformation (DLT) method with an approximately 2 m × 2 m × 2 m size radial calibration structure with 68 reference points and 5 reference markers set vertical and horizontal relative to the ground. The right-hand orthogonal reference frame was defined by the X_global_, Y_global_, and Z_global_ axes with the origin at the rear point of the home plate (Fig 1). The Y_global_ axis was directed from the home plate to the pitcher’s plate, and the Z_global_ axis indicated a vertically upward direction. The X_global_ axis was defined as the cross product of the Y_global_ and Z_global_ axes. For analysis of left-handed batters, a left-hand coordinate system with the same Y_global_ and Z_global_-axes as the right-hand coordinate system was utilized. To test the accuracy and reliability of this measurement method, one investigator digitized two reference markers on a swung bat for five frames on two separate occasions. The difference between the actual value (450 mm) and calculated value (mean ± standard deviation [*SD*] = 451 ± 0.3 mm). For the test-retest reliability of the distance, the coefficient of correlation was 0.973.

To clarify the spatial relationship between the bat’s sweet spot and the ball at the point of impact, in accordance with previous studies [16,25], the impact Z deviation and the impact X deviation (Fig 1) were calculated. First, the bat vector was defined as a vector lying along the long axis of the bat and oriented from the bat grip to the top. Then, the impact X deviation can be computed as

$${impact\;X\;deviation=b}_{x}∙(r_{Imp}-r_{SS}),$$ (1)

where *b*_*x*_ = unit vector that is parallel to the bat vector directed away from the sweet spot, *r*_*Imp*_ = position of the ball center at impact, and *r*_*SS*_ = position of the sweet spot at impact. The impact X deviation provides a measure of ball-bat contact in the direction parallel to the bat. The bat coordinate system was adopted to show the ball location on the bat barrel at the moment of contact. The impact Z deviation can be computed as

$${impact\;Z\;deviation=b}_{z}∙(r_{Imp}-r_{SS}),$$ (2)

where *b*_*z*_ = unit vector that is perpendicular to the bat vector directed upward in the vertical plane. The impact Z deviation provides a measure of ball-bat contact in the direction perpendicular to the bat.

### Statistical analysis

As an indicative factor of participants’ hitting ability, the speed of the bat head and sweet spot immediately before the ball-bat contact are expressed as the mean and SD. To verify the arrival location for all launched balls, the ball location at the moment of ball-bat contact in the global coordinate system was expressed as the mean and SD. The mean values of the impact X deviation and the impact Z deviation were analyzed to examine participants’ spatial component of systematic error in the hitting tasks. The mean ball location at ball-bat contact in the Y_global_ direction was analyzed to indicate the temporal aspect of participants’ hitting performance. The amount of systematic error for each direction was assumed to indicate how accurately a batter was able to decide where and when to swing the bat and control the bat’s barrel to the expected location and time of the arriving ball. These data were separately analyzed using two-way analysis of variance (ANOVA) with repeated measures at different ball speed settings (slow ball vs. fast ball) and occlusion conditions (*R+150* vs. *A-150* vs. *NO*) as within-subjects factors.

To examine the spatial component of participants’ hitting precision considering variability of the arrived ball location for each trial, standardized variability in the impact X deviation and impact Z deviation were calculated using standardized SDs for the impact X deviation and impact Z deviation with SDs for the arrived ball location in the X_global_ and Z_global_ directions. Therefore, values for standardized SDs of 1 indicated that SDs for the impact deviation were the same as SDs for the arrived ball location. Values for the standardized SDs became 0 when the locations of ball-bat contact were the same. Variability in the temporal aspect was quantified with the location of ball-bat contact in the Y_global_ direction to make it comparable to standardized variability in the impact X and Z deviations. The temporal variability of participants’ hitting performance was calculated using a standardized SD for the ball location at ball-bat contact in the Y_global_ direction with a SD for the expected ball location in the Y_global_ direction at the time of average ball flight time for all trials, with the measured ball speed of each trial. The expected ball location in the Y_global_ direction at the time of the average ball flight time of all the trials with the measured ball speed of each trial was used to evaluate variability in the position. Standardized variability in the three directions was analyzed using a three-way ANOVA with repeated measures using directions (impact X deviation vs. impact Z deviation vs. the ball location in the Y_global_ direction), speed settings, and occlusion conditions.

For all repeated measures factors with more than two levels, we tested whether Mauchly’s sphericity assumption was violated. If the result of Mauchly’s test was significant and the assumption of sphericity was violated, the Greenhouse-Geisser adjustment was used to correct the sphericity by altering the degrees of freedom using a correction coefficient epsilon. Significant results were further analyzed with a post hoc Bonferroni *t*-test for multiple comparisons. Statistical tests were performed using computer software (SPSS for Windows version 20.0; IBM SPSS, Tokyo, Japan). The α level of significance was set at *p* < 0.05.

## Results

### Bat swing speeds and ball arrival locations

Means and SDs of the speeds of the bat head and of the sweet spot in teed batting, which indicates the maximal bat speed, and the experimental conditions are shown in Table 1. The mean and SD of the ball location at the moment of ball-bat contact for each participant’s trials, which indicates the mean location and variability of the launched ball’s arrival location in the XZ_global_ plane, are shown in Table 2.

**Table 1:** Mean speeds (SD) of bat head and sweet spot (m/s) immediately before the ball-bat impact in tee batting and experimental conditions.

	bat head		sweet spot	
tee	34.0 (2.5)		27.6 (2.0)	
launched	slow ball	fast ball	slow ball	fast ball
*R+150*	32.4 (2.8)	32.5 (2.7)	26.2 (2.3)	26.3 (2.2)
*A-150*	32.7 (2.1)	32.4 (2.1)	26.5 (1.7)	26.2 (1.7)
*NO*	32.9 (1.9)	33.0 (2.3)	26.6 (1.5)	26.7 (1.9)

SD: standard deviation

**Table 2:** Mean and mean SD for each participant’s average ball arrival location in the global coordinate system (mm).

	slow ball				fast ball			
	X_global_		Z_global_		X_global_		Z_global_	
	Mean	SD	Mean	SD	Mean	SD	Mean	SD
R+150	117.6	48.0	816.0	77.7	100.7	50.6	833.0	74.0
A-150	118.8	47.1	825.6	72.6	110.1	52.8	846.2	72.4
NO	113.3	49.9	818.8	76.0	103.0	51.6	823.6	79.0

SD: standard deviation

### Ball flight duration and ball-bat contact locations

**Fig 2:**
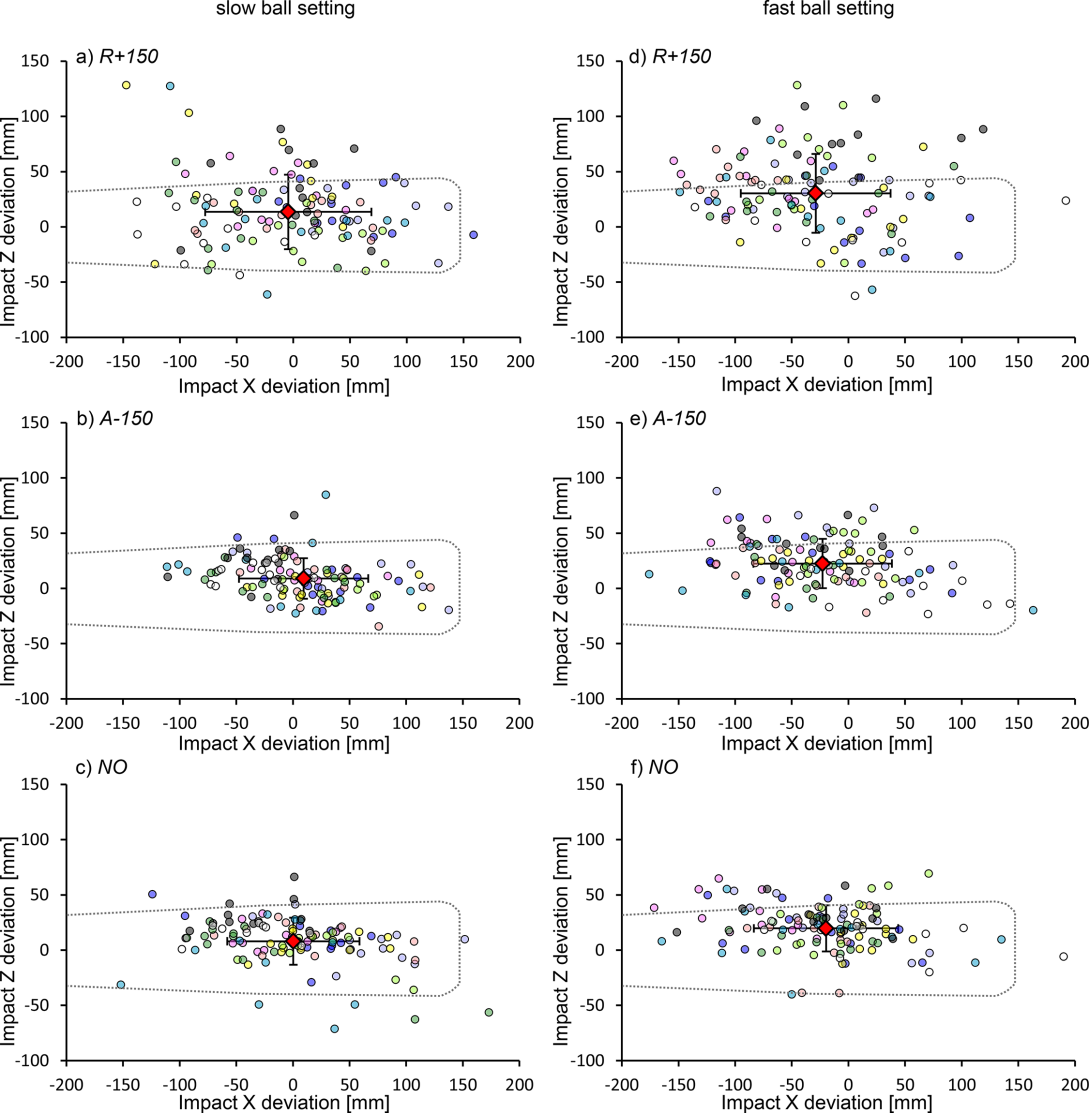
Scatter plot of the location of the ball center at the moment of ball-bat contact in all trials. The dashed line represents the barrel of the bat. The diamond symbol indicates the mean location of the distribution. The vertical and horizontal bars represent the mean SDs for the impact X and Z deviations for each participant. Each circle color corresponds to the data from one participant. (a) *R+150* condition in the slow ball setting. (b) *A-150* condition in the slow ball setting. (c) *NO* condition in the slow ball setting. (d) *R+150* condition in the fast ball setting. (e) *A-150* condition in the fast ball setting. (f) *NO* condition in the fast ball setting.

The mean ± SD of the ball flight time from ball release to ball-bat contact, or the moment the ball passed the bat, was 548.0 ± 3.6 ms in the slow ball setting and 453.3 ± 4.4 ms in the fast ball setting. The mean SDs for the expected ball location in the Y_global_ direction at the average ball flight time of all trials with the measured ball speed of each trial were 165.2 mm in the slow ball setting and 118.5 mm in the fast ball setting. The distributions of the ball center location at the moment of ball-bat contact, in the bat coordinate system, are shown in Fig 2.

### Systematic error in the ball-bat contact location

Two-way ANOVA for the mean values of the impact X deviation indicated no significant interaction and no main effect of ball speed or the occlusion condition. Two-way ANOVA for the mean values of the impact Z deviation showed a main effect of the ball speed setting (F_1.00, 9.00_ = 13.08, *p* < 0.01). A post hoc paired *t*-test showed that the impact Z deviation was significantly smaller in the slow ball setting than in the fast ball setting in the *A-150* (*t* [9] = -3.13, *p* < 0.05, *d* = -1.34) and *NO* (*t* [9] = -3.16, *p* < 0.05, *d* = -1.13) conditions. A post hoc multiple comparison test for the impact Z deviation for each ball speed setting indicated no significant difference.

**Fig 3:**
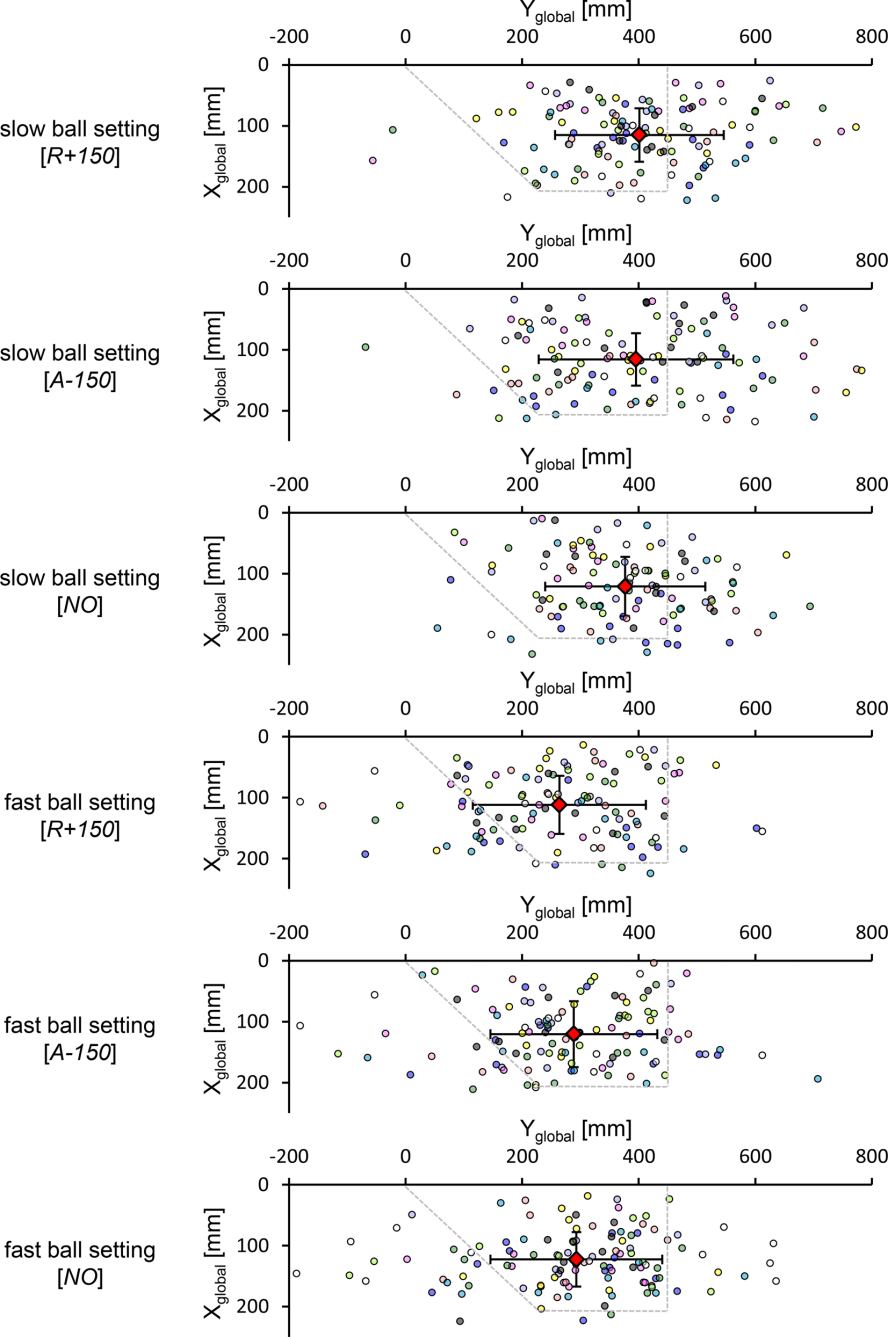
Ball locations in the slow ball setting and the fast ball setting for each visual occlusion condition (top view). The dashed line represents the outer half of a home plate for a right-handed batter. Each circle color indicates the ball locations at the moment of ball-bat contact for one participant. The diamond symbol represents the mean location of the distribution. The vertical and horizontal lines indicate the mean value of the SDs (i.e., the precision) of the ball locations at the moment of ball-bat contact for each participant.

For the temporal aspect of ball-bat contact, the mean ball location at the moment of ball-bat contact in the XY_global_ plane and the mean SD for the Y_global_ direction for each participant, which represents the temporal precision of ball-bat contact, are shown in Fig 3. Two-way ANOVA for the mean ball location in the Y_global_ direction showed a main effect of the ball speed setting (F_1, 9_ = 6.20, *p* < 0.05). However, this main effect was influenced by a significant interaction with the occlusion conditions (F_2, 18_ = 3.93, *p* < 0.05). A post hoc multiple comparison test for the mean ball location in the Y_global_ direction for each ball speed setting indicated no significant difference.

### Standardized variability of the ball-bat contact location

**Fig 4:**
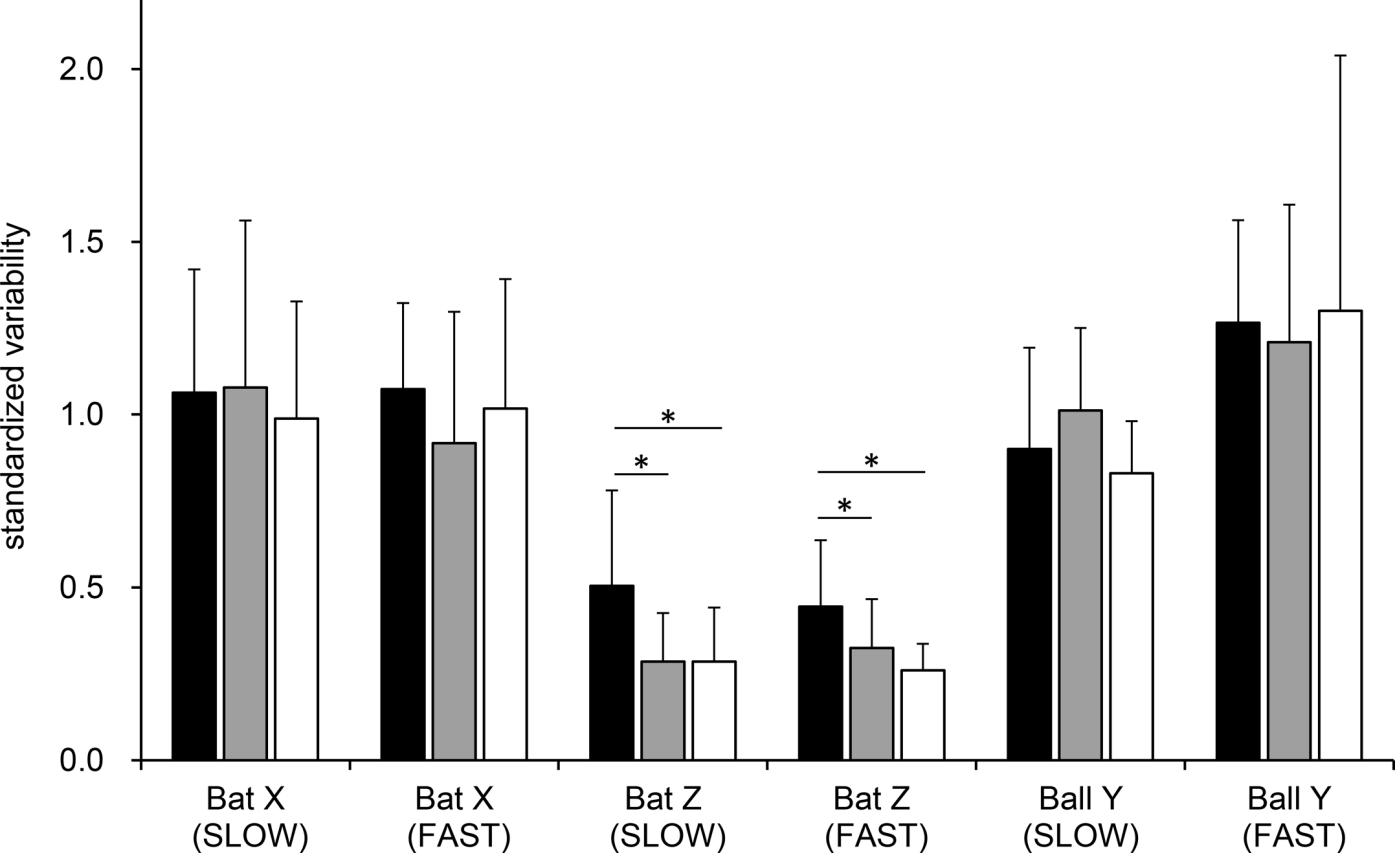
Standardized variability (mean + standard deviation) of the ball-bat contact location for each subject for the impact X deviation (Bat X), impact Z deviation (Bat Z), and timing (Ball Y) under the slow ball setting (SLOW) and fast ball setting (FAST). Variability for Bat X and Bat Z were calculated using standardized SDs for the impact X deviation and impact Z deviation with SDs for the arrived ball location in the X_global_ and Z_global_ directions. Variability for Ball Y was calculated using a standardized SD for the ball location at ball-bat contact in the Y_global_ direction with a SD for the expected ball location in the Y_global_ direction at the time of the average ball flight time of all the trials, with the measured ball speed of each trial. Each color of the bar represents an occlusion condition: black, *R+150*, gray, *A-150*, and white, *NO*. (*Standardized variability of the impact Z deviation under both ball speed settings was significantly larger in the *R+150* condition than in the *A-150* and *NO* conditions [*p* < 0.05]).

Three-way ANOVA for the variability of the ball-bat contact location showed a significant main effect of the directions of standardized variability (F_2,18_ = 54.94, *p* < 0.001) and a two-way interaction between the direction of standardized variability and the ball speed settings (F_2, 18_ = 4.38, *p* < 0.05). A post hoc multiple comparison test showed that the standardized variability of impact Z deviation was significantly smaller than the standardized variability of impact X deviation (*p* < 0.001) and ball-bat contact timing (*p* < 0.001). To further evaluate the effect of ball speed and occlusion, two-way ANOVAs for each direction of standardized variability were conducted separately. The results confirmed a significant main effect of occlusion (F_1.28, 11.45_ = 8.50, *p* < 0.05) in the variability of impact Z deviation. Post hoc multiple comparison tests for the variability of impact Z deviation under the slow ball and fast ball settings showed that the standardized variability of impact Z deviation was significantly larger under the *R+150* condition than under the *A-150* (*p* < 0.05) and *NO* (*p* < 0.05) conditions (Fig 4).

## Discussion

The hitting accuracy of college baseball batters under conditions of visual occlusion was examined to clarify the relationship between visual information and hitting accuracy. Based on the measured bat head speed right before ball-bat contact, which was equivalent to the bat head speed of professional baseball players measured in a previous study [1], the assumption that the swing time of participants in the present study was about the same as the previously reported swing time of professional baseball batters [2] was confirmed. Two ball launch speeds were used to reflect the range of ball speeds thrown by actual pitchers (31.9 m·s^-1^ [71.8 MPH] and 40.3 m·s^-1^ [90.7 MPH]). The location of ball-bat contact on the bat coordinate system and global coordinate system was analyzed to evaluate the participant’s hitting accuracy. In the present study, visual occlusion did not affect the mean location of ball-bat contact in the X_bat_, Z_bat_, and Y_global_ directions. However, standardized variability of the ball-bat contact location in the Z_bat_ directions was significantly larger during the *R+150* condition than during the *A-150* or *NO* condition. Thus, the hypothesis in the present study was supported only for the precision of ball-bat contact in the Z_bat_ direction. The lack of significant differences in the mean error and standardized variability of the impact Z deviation between the *A-150* and *NO* conditions suggests that visual information about the final one-third of the ball trajectory did not contribute significantly toward improving baseball hitting accuracy. As a pitched ball travels near the home plate, the angular velocity of eye movement required to track the ball exceeds the physiological limit of the human eye [12,26]. Although experienced batters possess a superior ability to track fast moving objects [12,27] and utilize head movement in addition to eye movement to track the ball [12,28], the final phase of ball movement may be too fast to track. In addition, because of the visuo-motor delay [7,8], batters may not be able to utilize the visual information about the ball trajectory right before the ball-bat contact, thus they must decide where to swing based on their prediction of the ball trajectory. The present study confirms that there is a directional specificity in the magnitude of variability with different visible time and a limitation in the effectiveness of visual information about ball trajectory for improving the hitting accuracy.

The standardized variability of ball-bat contact was significantly smaller in the Z_bat_ direction than in the X_bat_ and Y_global_ directions. Moreover, the mean location and standardized variability of the ball-bat contact in the X_bat_ and Y_global_ directions were not significantly different among all the occlusion conditions. A limitation of the present study was that the balls were launched toward only the strike zone above the outer half of the home plate from the batter, for participants’ safety. The small deviation of ball location in the X_global_ direction compared with the ball location in the Z_global_ direction (Table 2) may preclude adjustments to participants’ bat swing in the X_global_ direction. However, compared to a previous study that evaluated the performance of collegiate and semi-professional batters hitting baseballs off of a batting tee [25], the mean of the SDs for the X_bat_ direction was much greater in the present study (13 mm vs. 49.4 mm [Fig 2; *NO* in slow ball setting] or 53.3 mm [Fig 2; *NO* in fast ball setting]) while the mean of SDs for the Z_bat_ direction was only slightly greater in the present study (11 mm vs. 16.8 mm [*NO* in slow ball setting] or 19.2 mm [*NO* in fast ball setting]). Therefore, the experimental design of the present study seems to have augmented the error in the precision of ball-bat contact in both the Z_bat_ and X_bat_ directions. The lack of improvement in the impact X deviation as the occlusion duration became shorter can be explained by the properties of the bat or the influence of temporal error. The wider range of the effective impact area in the X_bat_ direction than the Z_bat_ direction, which can produce a fast-batted ball speed with an ideal vertical angle of projection, required participants to make less adjustment in the X_bat_ direction than the Z_bat_ direction. Although the definition and size of the “sweet spot” in the horizontal and vertical directions varies among scientists, the length in the vertical direction (approximately 10 mm to 25 mm) is commonly much smaller than the length in horizontal direction (approximately up to 100 mm) [5,6,23,24,28,29]. Temporal error of ball-bat contact likely increased the deviation of ball-bat contact in the X_bat_ direction. The ideal timing of ball-bat contact results in contact with the sweet spot of the bat. Due to the circular trajectory of a swung bat’s barrel in the transverse plane, if the ideal timing for ball-bat contact was involved in hitting the ball when the bat’s long axis is perpendicular to the pitcher-to-catcher direction, hitting a ball earlier than the ideal timing results in contact closer to the grip end of the bat, whereas hitting a ball later than the ideal timing of ball-bat contact results in contact closer to the barrel end of the bat. On the other hand, smaller deviation of ball-bat contact in the Z_bat_ direction can be explained by similar horizontal trajectory of a launched ball and a swung bat which may result in smaller vertical error of ball-bat contact due to the temporal error. Moreover, Brenner et al [30] reported that vertical precision for hitting a falling ball is improved by adjusting elevation of bat swing path. Therefore, several factors such as the property of the bat, temporal error, trajectory of a launched ball and a swung bat, and ability to adjust the swing height may lead to the difference of variability between the X_bat_ and Z_bat_ directions

The present study adopted two ball speeds, a relatively slow one and a relatively fast one, which are frequently observed in actual baseball games. A significant main effect of ball speed on the mean ball location in the Z_bat_ and Y_global_ directions may indicate that the faster ball speed in the fast ball setting forced participants to hit the ball farther from the ideal ball-bat contact location in the Z_bat_ and Y_global_ directions. In the *R+150* condition, although the length of the visible period was equal in the two speed settings, the slow ball setting resulted in a smaller impact Z deviation. The smaller impact Z deviation may be because of the longer time available to decide when and where to swing the bat. Therefore, the present result supports the idea that a shorter swing time and faster bat swing speed are beneficial for successful hitting because a longer time is available for making these decisions [31]. In addition, the lack of a significant difference in the precision of the impact Z deviation between the ball speed settings suggests that the preciseness of ball-bat contact may rely on visible time and perceptual ability and other factors such as accuracy in the execution of the bat swing motion to the intended location.

For the standardized variability of temporal accuracy, the lack of significant differences among the three conditions suggests that experienced batters can estimate the arrival time of the pitch by utilizing early visual information. Most variability may be due to the fast speed of the ball and bat, which travel a few millimeters in one millisecond, and there is also variability in swing onset and duration. A previous study of elite cricket batsman reported that they could hit the ball 60% of the time even when their vision was occluded from the moment of ball release [20]. However, unlike batting in a real baseball game, only one ball speed was used throughout a set of trials in the present study. This might allow the participants to hit the ball using the same timing repeatedly without visual information of either ball trajectory. In fact, the spatial and temporal error in ball-bat contact is greater when batters swing at a pitched ball with unexpected speed [32]. Another study reported that previous experience positively contributes to the accuracy of prediction for intercepting an occluded fly-ball trajectory [33]. The batter’s ability to predict the ball arrival time can be examined further by utilizing a task that requires hitting pitches at varying speeds.

In conclusion, the effect of occluding a batter’s vision for various periods of time on hitting accuracy was examined in the present study. The hypothesis that the longer the batter is able to see an approaching ball the more accurate their hitting will be unless visual occlusion occurs 150 ms before ball-bat contact was supported only by the precision of the ball-bat contact in the Z_bat_ direction. Although the magnitude of standardized variability in the Z_bat_ direction was significantly smaller than that in the X_bat_ and Y_global_ directions, additional visible time from the *R+150* condition to the *A-150* and *NO* conditions resulted in the further decrease in standardized variability in the Z_bat_ direction. The absence of significant differences between *A-150* and *NO* conditions suggests a limit in the effectiveness of such visual information about ball trajectory for improving hitting accuracy. The present study is the first to quantify baseball hitting accuracy with continuous data under different occlusion conditions, whereas previous studies evaluated hitting accuracy based on the type of contact (i.e. swing and miss, foul ball, ground ball, or fly ball). This study confirms that a longer time to see the ball and complete the bat swing results in a successful hit. In addition, similar to previous research on cricket batsmen, experienced baseball batters exhibited an ability to utilize visual information, especially during the early part of ball flight, to control bat location. For a future study, mixing fastballs with other types of pitches, such as curve balls, within a wider range of the strike zone will provide more generalizable knowledge of the relationship between visual information and hitting accuracy in baseball batters.
